# A cationic polymeric prodrug with chemotherapeutic self-sensibilization co-delivering MMP-9 shRNA plasmid for a combined therapy to nasopharyngeal carcinoma

**DOI:** 10.1080/10717544.2019.1698674

**Published:** 2019-12-03

**Authors:** Tao Liu, Xidong Wu, Shaohua Chen, Peina Wu, Hong Han, Hongbin Zhang, Junzheng Li, Guanxue Li, Siyi Zhang

**Affiliations:** a Department of Otolaryngology-Head and Neck Surgery, Guangdong Provincial People’s Hospital, Guangdong Academy of Medical Sciences, Guangzhou, China;; b Department of Drug Safety Evaluation, Jiangxi Testing Center of Medical Device, Nanchang, China;; c Department of Otolaryngology-Head and Neck Surgery, Guangzhou Red Cross Hospital, Medical College, Jinan University, Guangzhou, China;; d Department of Pediatric Center, Zhujiang Hospital of Southern Medical University, Guangzhou, China

**Keywords:** Chemotherapeutic sensibilization, polymeric prodrug, drug and gene co-delivery, nasopharyngeal carcinoma

## Abstract

To obtain a high-efficiency drug and gene co-delivery system to HNE-1 tumor therapy, a polymeric prodrug (PAAs-MTX) with chemotherapeutic sensibilization was synthesized consisting of a GSH-response hyperbranched poly(amido amine) (PAAs) and an antitumor drug of methotrexate (MTX). Then, the targeting molecule to HNE-1 cells, transferrin (Tf), was conjugated to form the Tf-PAAs-MTX. This polymeric prodrug could deliver MMP-9 shRNA plasmid (pMMP-9) again to form the drug and gene co-delivery system of Tf-PAAs-MTX/pMMP-9. The co-delivery system showed the effective drug and gene delivery ability with high cytotoxicity and gene transfection efficiency to HNE-1 cells. Besides that, Tf-PAAs-MTX also showed the chemotherapeutic sensibilization effect, the formulation containing PAAs segments showed much higher cytotoxicity than that of free MTX. Benefiting from the sensibilization effect and MTX/pMMP-9 co-delivery strategy, this Tf-PAAs-MTX/pMMP-9 co-delivery system exhibited the significantly improved therapeutic efficacy to HNE-1 tumor in a combined manner which was confirmed by *in vitro* and *in vivo* assays. Moreover, its biocompatibility, especially the blood compatibility was analyzed. This polymeric prodrug provided an easily delivery system combining the drug/gene co-delivery, chemotherapeutic sensibilization and targeting into one single platform, which showed a promising application in nasopharyngeal carcinoma therapy.

## 1. Introduction

Nasopharyngeal carcinoma (NPC) is one of the highest incidence tumors in South China (Zhou et al., 2019). In clinical, the radiotherapy and chemotherapy are the prevailing and effective strategies for NPC treatment. However, some side effects would limit their treatment effect, including the serious adverse effects, drug resistance and others (Ma et al., 2014; Liu et al., 2016; Liu et al., 2017). Nowadays, the drug and gene combined therapy has become the primary strategy in cancer and other disease therapy, because this technique would promote the synergistic actions, improve the target selectivity and deter the development of drug resistance. Some works have been reported on drug and gene co-delivery for cancer therapy, including NPC therapy (Zhou et al., 2016, 2018; Cui et al., 2019; Cheng et al., 2019; Lim et al., 2019). We also prepared some drug and gene co-delivery systems for NPC therapy and got some satisfactory results. For example, we used a glutathione (GSH)-response cationic polymer, the hyperbranched poly(amido amine)s (PAAs), to modify the graphene oxide (GO), the obtained PAAs-GO showed good drug and gene delivery ability to HNE-1 cells (Liu et al., 2019). However, GO showed strong interactions with the loaded drugs which resulted in the slow and insufficient drugs release; Moreover, the in vivo safety of GO is always a doubt in biomedicine.

Recently, the covalent polymeric prodrugs have become an important strategy to deliver effectively drugs, because polymeric prodrugs could increase and modulate the drug loading, improve the drug bioavailability, and enhance the cellular uptake due to the micelleization of the amphiphilic polymeric prodrugs (Cheng et al., 2019; Dong et al., 2019; Phua et al., 2019). So, a polymeric prodrug was synthesized in this work and then used to deliver plasmid for the drug and gene combined therapy to NPC.

To improve the therapeutic effects of the polymeric prodrugs to cancers, two strategies have been taken into consideration in this work. One is to conjugating a targeting molecule, transferrin (Tf), which has been reported to be targeted to NPC cells (Liu et al., 2018); the other is to use a chemotherapy self-sensibilized delivery system, which could enhance the chemotherapy effect of the prodrug. Some works including ours have confirmed that the GSH-response PAAs could be degraded in tumor cells due to the high intracellular GSH concentration, which means that PAAs could consume the intracellular GSH (Tang et al., 2018). The GSH-mediated detoxification is an important reason for the drug resistance of tumor cells. Therefore, the GSH-response PAAs could behave the chemotherapy sensibilization effect and then an enhanced therapy effect coming from the intracellular GSH consumption.

In this work, an antitumor drug, methotrexate (MTX), was conjugated with PAAs to form a polymeric prodrug PAAs-MTX, and then Tf was conjugated to form the Tf-PAAs-MTX. The therapeutic gene, MMP-9 shRNA plasmid (pMMP-9), closely related to tumor cells apoptosis and metastasis (Lin et al., 2017), was encapsulated through the electrostatic interaction between pMMP-9 and PAAs segments to form the Tf-PAAs-MTX/pMMP-9 complexes. In this work, we developed an “all-in-one” drug and gene co-delivery system comprised of sensitized chemotherapy, gene therapy as well as intracellularly GSH-triggered degradation and rapid drug/gene release, which would be useful in NPC therapy.

## 2. Experimental section

### 2.1. Materials

The polymeric prodrug of PAAs-MTX was kindly gifted by Dr. Tang who synthesized according to his previous work, and the MTX content in PAAs-MTX was determined to be 21 μg/mg by UV-Vis analysis (Tang et al., 2018). The Tf conjugation of the prodrug (Tf-PAAs-MTX) was synthesized according to our previous work (Liu et al., 2018). The branched polyethylenimine (PEI with a molecular weight of 25 kDa) were purchased from Sigma-Aldrich. All other reagents were purchased from Aladdin Industrial Corporation and used directly. A pcDNA3 plasmid vector constructed by LongSee-Med Technology Co., Ltd (Guangzhou) expressed the small interference RNA for MMP-9 protein with the enhanced green fluorescent protein (EGFP). Cell counting kit-8 (CCK-8) was obtained from Beyotime Institute of Biotechnology (Shanghai, China).Dulbecco’s Modified Eagle’s Medium (DMEM), Trypsine-EDTA (0.25%) and fetal bovine serum (FBS)were obtained from Gibco (BRL, MD, USA). The human nasopharyngeal carcinoma HNE-1 cells were supplied by Southern Medical University (China). Male specific pathogen-free BALB/c nude mice (age of 4 weeks, weight of 18–20 g) were obtained from the Center for Laboratory Animal Sciences, Southern Medical University (license number: SCXK (Yue) 2016-0041) and fed in the Experimental Animal Center of Jiangxi Testing Center of Medical Instruments (Jiangxi Institute of Materia Medica). The Institutional Administration Panel for Laboratory Animal Care approved the experimental design.

### 2.2. Sensitized chemotherapy to HNE-1 cells

The cytotoxicity of Tf-PAAs-MTX was evaluated by CCK-8 assay using HNE-1 and 3T3 cells. The HNE-1 cells were cultured onto a 96-well plate (5 × 10^3^ cells per well) in a complete DMEM (with 10% fetal bovine serum supplemented and high glucose) at 37 °C in a humidified atmosphere with 5% CO_2_. After overnight incubation, 100 µL complete DMEM that contained the desired amount of Tf-PAAs-MTX replaced the growth medium, and each sample was set for five multiple holes. The same amount of PBS was used as the control group. After 24 h incubation, 100 µL fresh DMEM with 10 µL of CCK-8 per wells were incubated in the cells for another 2 h. The absorbance at a wavelength of 450 nm in each well was measured by a microplate reader to calculate the number of viable cells. For PAAs sensitized MTX therapy assay, free MTX was also used the as control group. Moreover, in order to explore the targetability of Tf, Tf-PAAs-MTX and PAAs-MTX were incubated with HNE-1 cells and the cytotoxicity was determined by CCK-8 assay.

The intracellular GSH concentrations of HNE-1 cells treated with different formulations were determined by GSH quantification kit. Briefly, HNE-1 cells were seeded in a 24-well plate at a density of 5 × 10^4^ per well in complete DMEM and cultured overnight, and then treated with various formulations for another 24 h. After trypsinization, the cells were harvested and used for reduced GSH level measurement. The level of reduced GSH was measured by an enzymatic method according to the procedure from a commercial assay kit (Jiancheng Bioengineering Institute, Nanjing, China). Briefly, the reduced GSH could reacted with 5,5-dithio-bis (2-nitrobenzoic acid) (DTNB) to form a yellow compound, then the level of reduced GSH was measured at 405 nm over 5 min with a colorimetric assay.

### 2.3. pMMP-9 complexation

Tf-PAAs-MTX and pMMP-9 were co-dissolved in the distilled water with the suitable concentrations. Then, the resulting aqueous components were mixed at 25 °C, and gently stirred for 10 min to form the Tf-PAAs-MTX/pMMP-9 complexes. Their particle sizes and zeta potentials were recorded by a dynamic light scattering (DLS, Zetasizer Nano-ZS, Malvern, United Kingdom) with a monochromatic coherent He-Ne laser, and their morphologies was observed by a transmission electron microscope (TEM, JEM-2010HR, Japan).

The binding ability of Tf-PAAs-MTX to pMMP-9 was evaluated by gel electrophoresis assay. TAE buffer (1 mmol/L EDTA and 40 mmol/L Trisacetate) was used to prepare the agarose gel (1.0%, w/v) containing the ethidium bromide (0.25 mg/mL). After an incubation for 15 min at 25 °C, all samples were performed by electrophoresis on the agarose gel at 70 V for 20 min. Visualization and image capture were accomplished using the UV-trans illuminator under a Kodak EDAS 290 digital imaging suite (Fisher Scientific, PA).

### 2.4. *In vitro* transfection

HNE-1 cells were seeded into the 24-well culture plates using 500 µL complete DMEM as the culture medium at a density of 4 × 10^4^ cells per well. After 12 h incubation, the culture medium was interchanged by fresh Tf-PAAs-MTX/pMMP-9 complexes (weight ratios of from 20 to 80) in Opti-DMEM. The pMMP-9 amount in each well was fixed at 2.0 µg. The cells were incubated for another 36 h and then analyzed by green fluorescent protein (GFP) expression using a fluorescence microscope (Nikon-2000U, Japan). The cells treated with PEI-25k/pMMP-9 (weight ratio of 1.3) and Tf-PAAs/pMMP-9 complexes were set as the control groups. After the cells were digested by trypsinase, the transfection percentages (positive cell percent) were recorded by a flow cytometer (BD Accuri C6).

For transwell assay, HNE-1 cells were seeded in a 24-well plates at a density of 5 × 10^4^ cells per well in complete DMEM and incubated overnight. Cells were replenished with Opti-MEM (without serum) containing various formulations, and PEI-25k/MMP-9 (w/w = 1.3) was set as control. After 6 h incubation, the cells was replenished with fresh culture medium and incubated for another 24 h. The transfected cells were plated (2 × 10^4^ cells/well) onto the upper compartment of the transwells. Another 24 h later, the cells detained in the upper wells were removed and the cells that had passed through the membrane on the lower surface of the insets were fixed and stained using crystal violet. The migration result was observed with microscope and quantified with Image Pro Plus 6.0 software.

### 2.5. Combined therapy

#### 2.5.1. Cytotoxicity

HNE-1 cells were cultured into a 96-well plate (8 × 10^3^ cells/well) in complete DMEM at 37 °C in a humidified atmosphere with5% CO_2_. After 12 h incubation, the medium was replaced by 100 µL of complete DMEM containing the desired amount of formulations, and every formulation was set for five multiple holes. Cells treated with the same amount of PBS and Tf-PAAs were used as the control groups. After 24 h incubation, the cells were incubated in 100 μL of DMEM containing CCK-8 for another 2 h. The absorbance in each well was measured at a wavelength of 450 nm to calculate the number of viable cells.

#### 2.5.2. Apoptosis

HNE-1 cells were seeded in 24-well plates at a density of 5 × 10^4^ cells per well in complete DMEM and incubated overnight. The medium was then renewed by the medium containing various formulations respectively. Cells treated with PBS were applied as the control. After 6 h treatment, the formulations were replaced by fresh culture medium and incubated for another 24 h. Then the cells were immediately trypsinized, collected and resuspended in 200 µL of binding buffer. Afterwards, 5 µL of Annexin V-PE and 10 µL of 7-ADD were added and kept in the dark for 15 min. The stained cells were analyzed using flow cytometer (BD Accuri C6).

#### 2.5.3. *In vivo* assays

For *in vivo* anti-tumor tests, the HNE-1 tumor-bearing nude mice were modeled and then randomly divided into five groups (*n* = 5). Subsequently, the mice were injected intravenously using 200 μL Tf-PAAs-MTX/pMMP-9 (pMMP-9 dose of 5 μg/g and MTX dose of 6.56 μg/g). PBS (200 μL), Tf-PAAs-MTX (6.56 μg/g MTX in 200 μL PBS), Tf-PAAs-MTX/pMMP-9 (pMMP-9 dose of 5 μg/g in 200 μL PBS), and blank Tf-PAAs (0.3 mg/g in 200 μL PBS) were set as the control groups. Particularly, all formulations were injected every two days. After 3 weeks, the animals were sacrificed. The tumor volume was calculated as: V = (L × W^2^)/2, where W and L denote the shortest and longest diameters of the tumors respectively.

### 2.6. Biocompatibility

#### 2.6.1. Hemolysis

For hemolysis testing, the fresh whole blood was taken from mice using the sodium citrate as an anti-coagulant with a blood/anticoagulant ratio of 9:1. The whole blood was immediately centrifuged at 1000 rpm for 5 min to remove the resultant buffy coat layer and plasma. The obtained red blood cells (RBCs) were washed with PBS (pH = 7.4) for three times. After that, the RBCs were re-suspended in PBS at 16% hematocrit (v/v) and then mixed with 5 mL PBS containing Tf-PAAs-MTX at different concentrations (1, 10, 100 and 1000 μg/mL respectively) for 12 h. The positive (100% hemolysis induced by PBS containing 5 mL 0.1% Na_2_CO_3_ solution) and negative (0% hemolysis of only PBS) controls were set up. Each sample was measured for three times. After the incubation, the RBC suspensions were centrifuged at 1000 rpm for 5 min, and the supernatants were measured for the absorbance at 540 nm. The percentage hemolysis was calculated by the optical density (OD) values as the following formula (Zhang et al., 2019a):

Hemolysis (%)=[(OD of the test sample–OD of negative control)×100]  /OD of positive control.

#### 2.6.2. Activated partial thromboplastin time (APTT) and prothrombin time (PT) assays

The APTT and PT assays were recorded by the SF-8000 automatic coagulation analyzer (Beijing Succeeder Company, China) with the corresponding reagents provided by the Southern Medical University (Guangzhou, China). Platelet-poor plasma was obtained by centrifuging the citrated whole blood at 1000 rpm for 15 min and then mixed with Tf-PAAs-MTX at different concentrations (1, 10, 100 and 1000 μg/mL respectively). Then, APTT and PT were analyzed at 37 °C. Each experiment was repeated for three times. The sample of platelet-poor plasma mixing with only PBS was set as control.

#### 2.6.3. Thromboelastography (TEG) assay

The blood coagulation process in the presence of Tf-PAAs-MTX was studied at 37 °C using TEG hemostasis system 5000 (Haemoscope Corporation, USA). 360 μL of citrate-anticoagulated whole blood was mixed with 40 μL of aqueous Tf-PAAs-MTX solution in a tube containing kaolin, and the final concentration of HPG-C18-PLLD was from 1 to 1000 μg/mL. Then the samples were transferred into the TEG cup, and the analysis was initiated by the addition of 20 μL aqueous 0.2 mol/L CaCl_2_. PBS was used as the control.

#### 2.6.4. Cytotoxicity to 3T3 cells

The cytotoxicity of Tf-PAAs and Tf-PAAs-MTX to 3T3 cells were determined by CCK-8 assay *in vitro*. The 3T3 cells were cultured onto a 96-well plate (7 × 10^3^ cells per well) in complete DMEM (with 10% fetal bovine serum supplemented and high glucose) in a humidified atmosphere of 5% CO_2_ at 37 °C for 12 h. The growth medium was then replaced with 200 µL DMEM containing the desired amount of formulations, and five multiple holes were set for every sample. The PBS was used as the control group. After 24 h cultivation, 100 µL of DMEM with 10 µL of CCK-8 were added and then incubated for another 2 h. The cell viability was determined at a wavelength of 450 nm by a microplate reader.

#### 2.6.5. *In vivo* toxicity

For *in vivo* toxicity study, the above nude mice after 21 days *in vivo* assays were sacrificed, and their major organs (heart, liver, spleen, lung and kidney) were harvested and washed with PBS. After fixed by 4% formaldehyde, histological examination was carried out.

### 2.7. Statistical analysis

All data were expressed as the mean ± the standard deviation. GraphPad Prism 5 (GraphPad Software, Inc., La Jolla, CA, USA) was used to perform statistical analysis (one-way analyses of variance, ANOVA). The significance level was 0.05, and the data were indicated with * for *p* < 0.05, ** for *p* < 0.01 and *** for *p* < 0.001.

## 3. Results and discussions

### 3.1. Sensitized chemotherapy

**Scheme 1.**
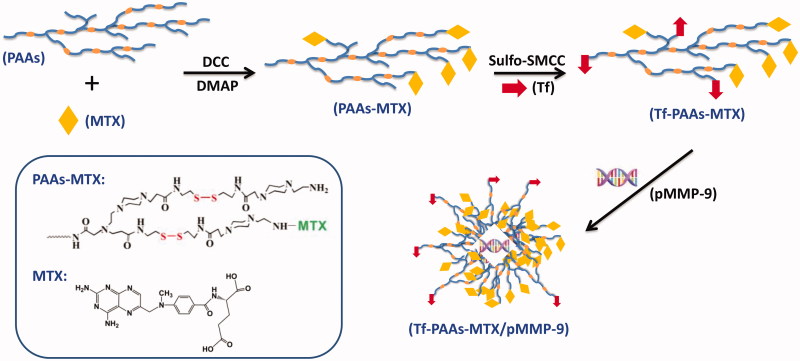
The schematic diagram of Tf-PAAs-MTX/pMMP-9 complexes preparation.

As shown in Scheme 1, to obtain a high-efficiency delivery system with the self-sensible chemotherapy and gene therapy, the GSH-response PAAs was firstly synthesized according to the reported work (Tang et al., 2018), and then MTX and Tf were conjugated in turn with PAAs through the chemical reaction. Particularly, the Tf containing mercapto group was conjugated with the amino group of PAAs to form the Tf-PAAs-MTX carrier using the sulfo-SMCC as the activity. The obtained Tf-PAAs-MTX could co-deliver pMMP-9 through the electrostatic interactions between PAAs segment and pMMP-9. The MTX and Tf content in Tf-PAAs-MTX was determined to be 2.1 wt% and 1.3 wt% respectively, which were analyzed by the UV-Vis spectrophotometer and Micro BCA Protein Assay Kit.

**Figure 1.**
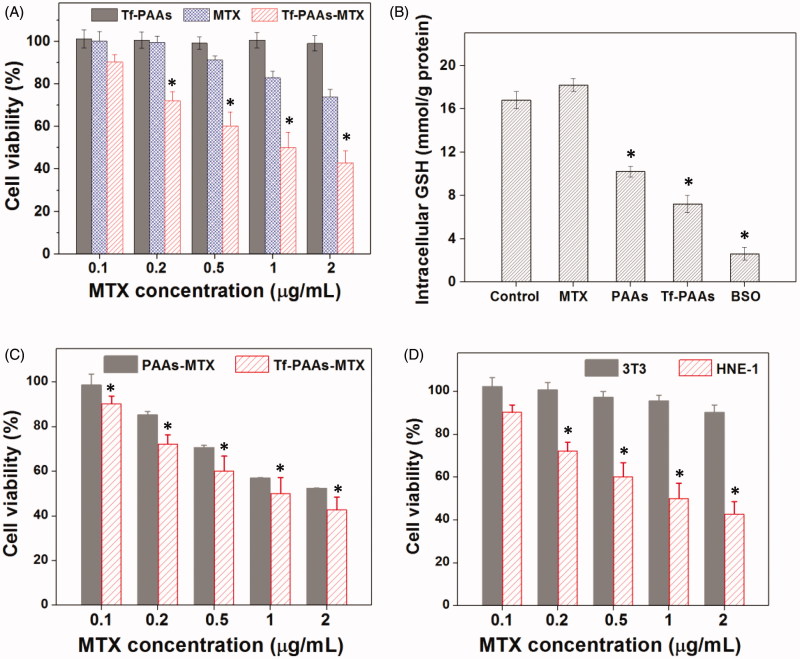
Sensitized chemotherapy to HNE-1 cells. (A) HNE-1 cells viability after incubated with blank Tf-PAAs, free MTX and Tf-PAAs-MTX for 24 h (37 °C and 5% CO_2_, *n* = 5). (B) Intracellular GSH of HNE-1 cells treated with different formulations (*n* = 5). (C) HNE-1 cells viability after incubated with Tf-PAAs-MTX and PAAs-MTX (37 °C and 5% CO_2_, *n* = 5). (D) Cytotoxicity of Tf-PAAs-MTX to HNE-1 cells and 3T3 cells (37 °C and 5% CO_2_, *n* = 5).

PAAs has been confirmed to be degraded under a high GSH concentrations, which makes it an enhanced chemotherapy effect to tumor cells by consuming their intracellular GSH expect of a high-efficiency drug/gene delivery (Chen et al., 2019). Tang et al have reported that the PAAs segments could be degraded intracellularly and consume the intracellular GSH, and then the GSH expression could be down-regulated (Tang et al., 2018). The down-regulated GSH could improve the sensibility of some tumor cells to chemotherapy drugs, and this strategy has been used in clinical tumor therapy. So, in this work, the drug carrier containing PAAs segments could improve the sensitivity of HNE-1 cells to MTX and showed the self-sensibilization effect of the drug carrier. Moreover, compared with traditional drug delivery systems such as micelles, the polymeric prodrug showed an advantage in modulating drug loading and bioavailability (Dong et al., 2019). To confirm these, the *in vitro* HEN-1 cells inhibition assays were performed. Figure 1(A) showed the results of HNE-1 cells viability after incubated with blank Tf-PAAs, free MTX and Tf-PAAs-MTX for 24 h. It was found that blank Tf-PAAs showed non-cytotoxicity to HNE-1 cells in the experimental concentrations, indicating a good biocompatibility of this drug delivery system. Free MTX and Tf-PAAs-MTX showed the obvious cytotoxicity to HNE-1 cells with the concentration dependence. In particular, the polymeric prodrug of Tf-PAAs-MTX showed significantly better inhibition effect on HNE-1 cells than that of free MTX. For example, the Tf-PAAs-MTX showed the inhibition efficiency of more than 40% to HNE-1 cells at the MTX concentration of 0.5 µg/mL, at which the free MTX showed only less than 10% inhibition efficiency. According to the inhibition results, the IC50 of the free MTX and Tf-PAAs-MTX to HNE-1 cells were determined as 7.65 µg/mL and 0.93 µg/mL (concentration of MTX) respectively, implying that Tf-PAAs-MTX displayed much better therapeutic effect than free MTX to HNE-1 tumor, which may be relative to the micelleization of the polymeric prodrug and the self-sensibilization effect of PAAs segment. Compared with free MTX, the polymeric prodrug of Tf-PAAs-MTX showed an amphiphilic property and then formed the micelles in aqueous solutions due to the hydrophilic PAAs and hydrophobic MTX. These micelles easily interacted with cells due to its cationic surface which could interact with the anionic cell surface, as well were uptaken by the cells due to the amphiphilic property of the cell membrane (Fu et al., 2019). Moreover, intracellularly, PAAs segments could be degraded easily by GSH, which was overexpressed in HNE-1 cells (Huang et al., 2019). GSH mediated detoxification has been reported as one of the most important mechanisms responsible for the cancer drug resistance (Zhao et al., 2019). As an antioxidant *in vivo*, GSH prevents the damage of cellular components caused by chemical substances including heavy metals and drug metabolites, etc (Li et al., 2019). The GSH levels in tumor cells are comparatively higher than those in normal cells, which suggested that the intracellular GSH excessive consumption may alleviate the resistance of cancer cells to the chemotherapeutic agents (Wu et al., 2019). For Tf-PAAs-MTX, the intracellular degradation of PAAs segment consumed the GSH and then improved the sensibility of HNE-1 cells to the drug of MTX. To confirm this, the intracellular GSH concentration of HNE-1 cells treated with different formulations were tested and the results were shown as Figure 1(B). It was found that free MTX could not down-regulate the GSH concentration intracellular HNE-1 cells, which showed no significant difference with PBS control. The BSO (buthionine sulfoximine), which can selectively inhibit the synthesis of GSH and further influence the GSH-mediated detoxification process, could down-regulate significantly GSH concentration and has been used as a drug sensitizer in combination therapy with chemotherapeutic agent in clinical (Song et al., 2019). For PAAs and Tf-PAAs, the intracellular GSH concentrations of HNE-1 cells decreased obviously, indicating that PAAs segment could down-regulate the GSH express and then improved the sensibility of HNE-1 cells to the chemotherapy drugs. Moreover, Tf-PAAs showed more down-regulation of GSH than PAAs, which may be attributed from the Tf-targeting effect to HNE-1 cells.

To further verify the targeting ability of Tf to HNE-1 cells, the cells were incubated with Tf-PAAs-MTX and PAAs-MTX, and the cell viability results were shown as Figure 1(C). It was found that both Tf-PAAs-MTX and PAAs-MTX showed the concentration-dependent cytotoxicity to HNE-1 cells and the Tf-PAAs-MTX showed significantly better inhibition effect than PAAs-MTX. This result confirmed that Tf was targeting to HNE-1 cells and Tf-PAAs-MTX could be uptaken by HNE-1 cells easier than PAAs-MTX.

In this work, Tf-PAAs-MTX showed the excellent HNE-1 cell inhibition through its self-sensibilization effect due to the overexpressed GSH in HNE-1 cells. Then, the normal cells without overexpressed GSH, such as 3T3 was also be tested. The cell viabilities of both HNE-1 cells and 3T3 cells were performed and shown in Figure 1(D). It was found that there was significant difference in cell viability between HNE-1 cells and 3T3 cells treated with Tf-PAAs-MTX at the same concentrations, and Tf-PAAs-MTX displayed no inhibition effect to 3T3 cells. This result further verified our assumption that the excellent inhibition to HNE-1 cells of Tf-PAAs-MTX was attributed to its GSH-response property. The overexpressed GSH in HNE-1 cells made Tf-PAAs-MTX degrade and then release MTX, meanwhile GSH was consumed and the GSH-mediated detoxification process was reduced. However, in 3T3 cells, PAAs segment degraded much slowly and MTX was released little. Such a chemical structure of introducing abundant disulfide bonds in PAAs backbone made the Tf-PAAs-MTX prodrug the high-efficiency chemotherapy to HNE-1 cells.

### 3.2. pMMP-9 delivery

**Figure 2.**
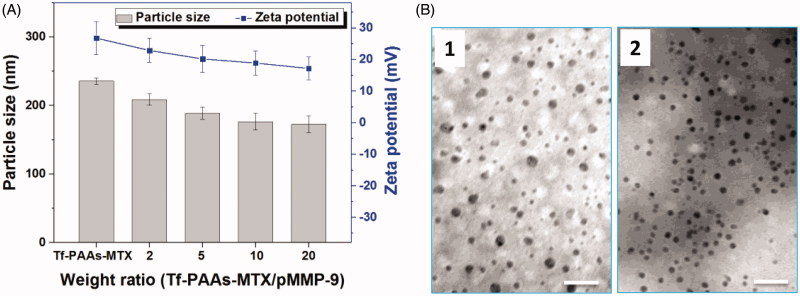
(A) The zeta potentials and particle sizes of Tf-PAAs-MTX/pMMP-9 complexes with different weight ratios (*n* = 5). (B) TEM images of Tf-PAAs-MTX and Tf-PAAs-MTX/pMMP-9 (w/w = 20).

Tf-PAAs-MTX has been developed as an efficient gene delivery vector benefitting from its hyperbranched cationic PAAs segment. To confirm the pMMP-9 delivery ability of Tf-PAAs-MTX, particle size and zeta potential were tested first. As shown in Figure 2(A), Tf-PAAs-MTX showed a particle size of 235 nm and zeta potential of +25 mV due to its micelleization. From the TEM image shown in Figure 2(B), Tf-PAAs-MTX micelles showed the compact spherical morphology. After binding pMMP-9 through the electrostatic interaction, the Tf-PAAs-MTX/pMMP-9 complexes showed a smaller but more uniform spherical morphology, implying that Tf-PAAs-MTX could bind pMMP-9 effectively and form the compact structure. Moreover, the positive zeta potentials of Tf-PAAs-MTX/pMMP-9 complexes ensured that the complexes could be uptaken by cells and then pMMP-9 could be delivered into cells.

**Figure 3.**
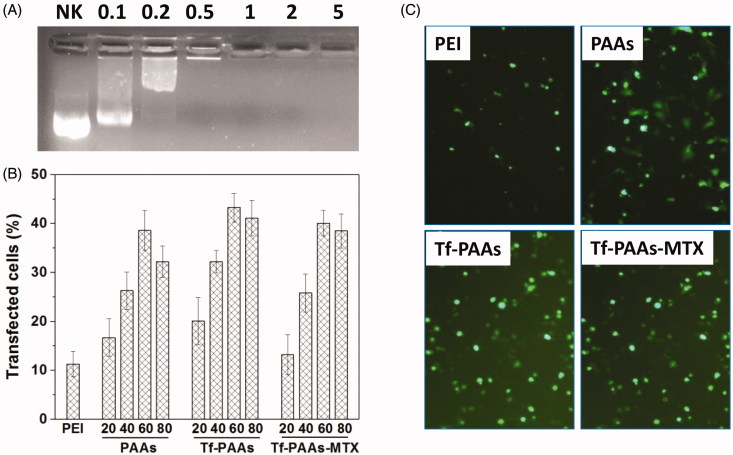
(A) Gel electrophoresis assay of Tf-PAAs-MTX/pMMP-9 complexes with different weight ratios. (B) Gene transfection results of HNE-1 cells treated with PEI-25k/pMMP-9 (w/w = 1.3), PAAs/pMMP-9, Tf-PAAs/pMMP-9 and Tf-PAAs-MTX/pMMP-9 (w/w = 20, 40, 60 and 80 respectively) complexes (*n* = 3). (C) Typical image of transfected HNE-1 cells with PEI-25k/pMMP-9 (w/w = 1.3), PAAs/pMMP-9 (w/w = 60), Tf-PAAs/pMMP-9 (w/w = 60) and Tf-PAAs-MTX/pMMP-9 (w/w = 60).

The gel retardation assay was also performed to examine its gene condensation ability. As shown in Figure 3(A), at the low Tf-PAAs-MTX/pMMP-9 weight ratios of 0.1:1–0.5:1, pMMP-9 could be observed to move out under the experimental electric field conditions, indicating that Tf-PAAs-MTX/pMMP-9 could not bind pMMP-9 entirely. Above the ratio of 1:1, pMMP-9 was totally retarded, implying that the pMMP-9 was completely condensed by Tf-PAAs-MTX. Such a low weight ratio of Tf-PAAs-MTX totally retarding pMMP-9 also suggested that Tf-PAAs-MTX showed the good gene condensation ability *in vitro*.

The gene transfection efficiencies of Tf-PAAs-MTX/pMMP-9 complexes at different weight ratios was evaluated by *in vitro*, and PEI-25k/pMMP-9 (w/w = 1.3) and PAAs/pMMP-9 as well as Tf-PAAs/pMMP-9 were used as the controls. As shown in Figure 3(B,C), formulations containing PAAs segments showed the considerable gene delivery ability to pMMP-9, while PEI-25k showed the poor transfection efficiency due to its unstability in the presence of serum. Their high transfection efficiency may arise from two reasons: one is that PAAs segment displayed the good blood compatibility, which makes their complexes more stable in the serum; the other is that its hyperbranched structure and bioreducible disulfide linkages could improve the local cationic charge density as well as release gene rapidly intracellularly. Particularly, Tf-PAAs showed significant higher transfection efficiency than PAAs, confirming the good targeting ability of Tf to HNE-1 cells. At the weight ratio of 60, Tf-PAAs showed the best transfection ability in this work, and about 43% HNE-1 cells could be transfected. Moreover, Tf-PAAs-MTX showed a little decrease in transfection efficiency compared with Tf-PAAs, which may be attributed to the cytotoxicity of MTX, affecting HNE-1 cell status when transfecting.

### 3.3. Combined therapy

**Figure 4.**
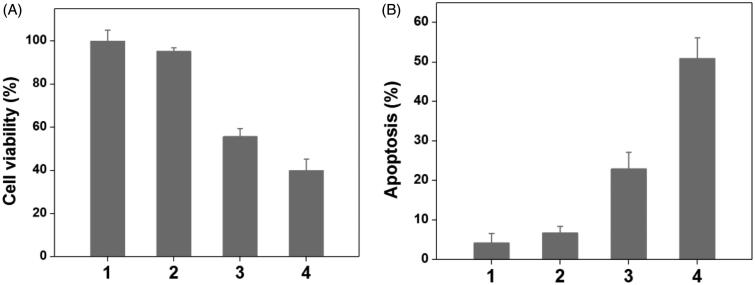
The HNE-1 cell viability and apoptosis results treated with different formulations (*n* = 5), (1: PBS; 2: blank Tf-PAAs; 3: Tf-PAAs-MTX; 4: Tf-PAAs-MTX/pMMP-9).

To verify the drug/gene co-delivery strategy for HNE-1 tumor therapy, *in vitro* CCK-8 assay was firstly carried out to verify the combined inhibition effect of pMMP-9 and MTX. As shown in Figure 4(A), the cells treated with blank Tf-PAAs showed the negligible inhibition effect on HNE-1 cells, indicating the good biocompatibility as the drug/gene delivery system. The formulation of Tf-PAAs-MTX showed the significant cytotoxicity to HNE-1 cells due to the enhanced chemotherapy effect as expected. For the further delivery of pMMP-9, Tf-PAAs-MTX/pMMP-9 displayed the best inhibition effect and more than 60% HNE-1 cells were inhibited, significantly higher than that of only MTX used. Figure 4(B) showed the apoptosis effect of HNE-1 cells treated with different formulations and got the similar results, of which the co-delivery strategy showed the highest apoptosis ratio with more than 50%. These results indicated that the drug/gene co-delivery strategy displayed the outstanding inhibition effect on HNE-1 cells and had the potential application in combined tumor therapy.

**Figure 5.**
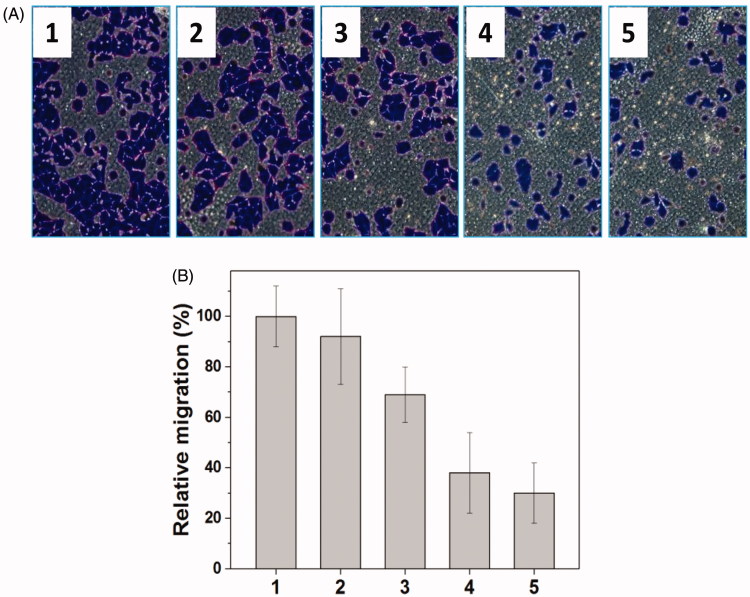
Representative images of HNE-1 cells traversed transwell-chamber (A) and the relative migration rates (B) after incubated with various formulations (1: PBS; 2: Tf-PAAs; 3: Tf-PAAs-MTX; 4: Tf-PAAs/pMMP-9; 5: Tf-PAAs-MTX/pMMP-9) (*n* = 3).

Metastasis-related recurrence responsible for the majority of the mortality is a common occurrence in HNE-1 cancer, which is similar to most reported tumor diseases (Yang et al., 2006), so the target to the inhibition of migration-related signals, MMP-9 shRNA plasmid, was delivered in this work, and the transwell assay was carried out to examine the migration of HNE-1 cells treated with various formulations. As shown in Figure 5(A,B), the blank Tf-PAAs showed no evident effect on migration ability of HNE-1 cells compared with PBS control, while Tf-PAAs/pMMP-9 caused the obvious migration capability suppression efficiently. This result implied that the down-regulation of MMP-9 protein could inhibit effectively HNE-1 cells migration. Moreover, Tf-PAAs-MTX also showed the obvious migration suppression to HNE-1 cells, indicating the cytotoxicity of MTX affected HNE-1 cell viability significantly. Then, the co-delivery system of Tf-PAAs-MTX/pMMP-9 displayed the most significant result of suppressing HNE-1 cells migration, suggesting that the strategy of co-delivery of drug and gene could efficiently inhibit HNE-1 cell migration.

**Figure 6.**
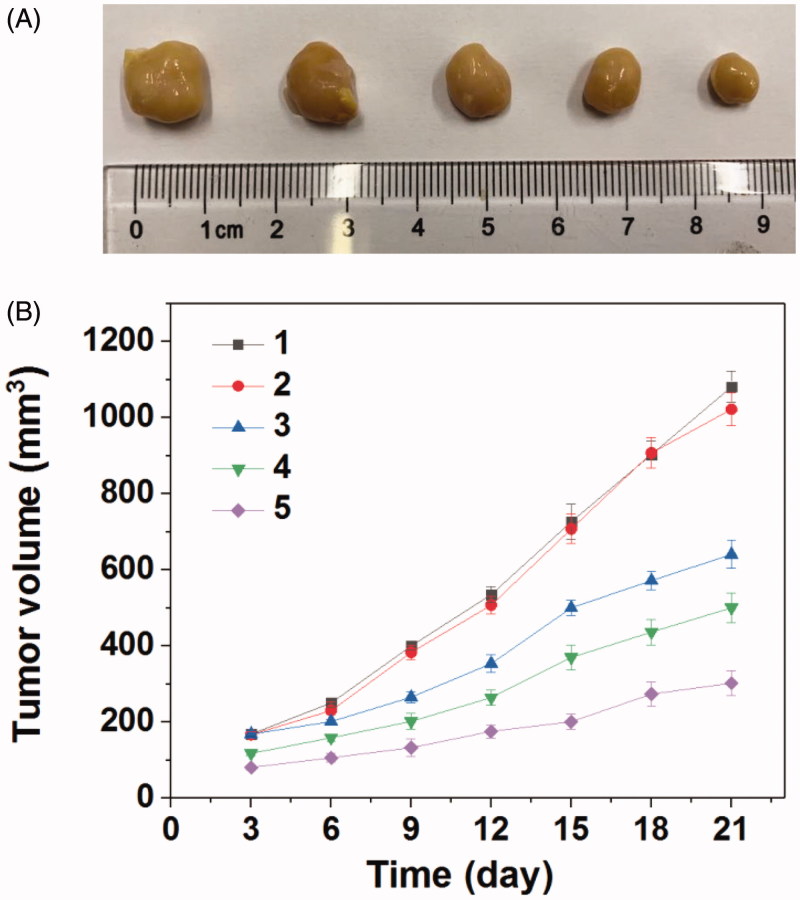
(A) Representative image of HNE-1 tumors after the 21 days and (B) tumor growth profiles treated with different formulations (*n* = 5) (1: PBS; 2: Tf-PAAs (0.3 mg/g); 3: Tf-PAAs/pMMP-9 (pMMP-9 dose of 5 μg/g); 4: Tf-PAAs-MTX (MTX dose of 6.56 μg/g); 5: Tf-PAAs-MTX/pMMP-9 (pMMP-9 dose of 5 μg/g and MTX dose of 6.56 μg/g)).

Encouraged by the good combined therapeupic effect *in vitro*, the *in vivo* antitumor assay was then investigated. Figure 6 gave the tumors growth profiles and the representative tumors image treated with various formulations. It was found that the blank Tf-PAAs showed no inhibition effect on HNE-1 tumor and there was no significant difference in growth profiles with PBS control. The other groups treated with MTX or pMMP-9 formulations displayed the obvious antitumor effect. Particularly, the tumors treated with Tf-PAAs-MTX/pMMP-9 showed the best antitumor effect with the smallest tumor volume and slowest tumor growth profile, which was a significant difference with other groups. This result indicated that the drug and gene co-delivery system as well as the self-sensibilization effect showed the potential application in combined tumors therapy.

### 3.4. Biocompatibility

**Figure 7.**
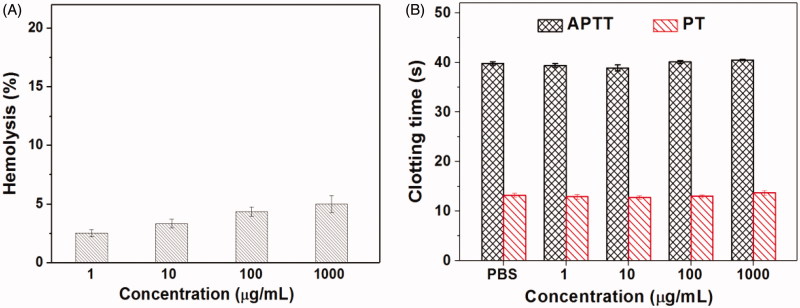
The hemolysis (A) and clotting analysis (B) of Tf-HPAA-MTX/pMMP-9 (*n* = 3).

For the drug delivery systems, their blood compatibility is a must concern. Their interactions with the compositions of blood are considered as the serious limitation in clinical, and their nonspecific interactions could even severely diminish the half-life and targeting of drugs (Xiao et al., 2019). The blood compatibility of Tf-PAAs-MTX/pMMP-9 was assessed by spectrophotometric measurement of hemoglobin released from erythrocytes after Tf-PAAs-MTX/pMMP-9 treatment. Figure 7(A) showed the percentage hemolysis of the blood in contact with different Tf-PAAs-MTX/pMMP-9 concentrations. It was found that Tf-PAAs-MTX/pMMP-9 exhibited the good blood compatibility. Even the concentration reached to 1 mg/mL, the sample showed a non-hemolytic effect with the extent of hemolysis lower than the permissible level of 5% (Bi et al., 2019).

Another important concern on blood compatibility for drug carriers is the effect on the blood coagulation (Zhang et al., 2019b). Coagulation at the right time and location is necessary to maintain normal metabolism, while inappropriate coagulation will cause severe, evenfatal, risks to the living system. Therefore, the effect of Tf-PAAs-MTX/pMMP-9 on coagulation is a key factor in the blood safety evaluation. The blood coagulation cascade contains three types of pathways: intrinsic, extrinsic and common pathway. Thereinto, the performance of the intrinsic and common coagulation pathways are measured by APTT, which refers to the time needed for forming a fibrin clot after a partial thromboplastin reagent or CaCl_2_ is added. Meanwhile, PT measures the performance of both extrinsic and common coagulation pathways, and refers to the time taken to form a fibrin clot after tissue thromboplastin is added. The effects of the Tf-PAAs-MTX/pMMP-9 on APTT and PT are shown in Figure 7(B). Compared with the PBS control, Tf-PAAs-MTX/pMMP-9 did not significantly change the APTT and PT of the blood under the concentration of 1 mg/mL. The results indicated that Tf-PAAs-MTX/pMMP-9 under experimental concentrations had no obvious activation to coagulation factor XII in the plasma and thrombin, suggesting the blood safety in this work.

**Figure 8.**
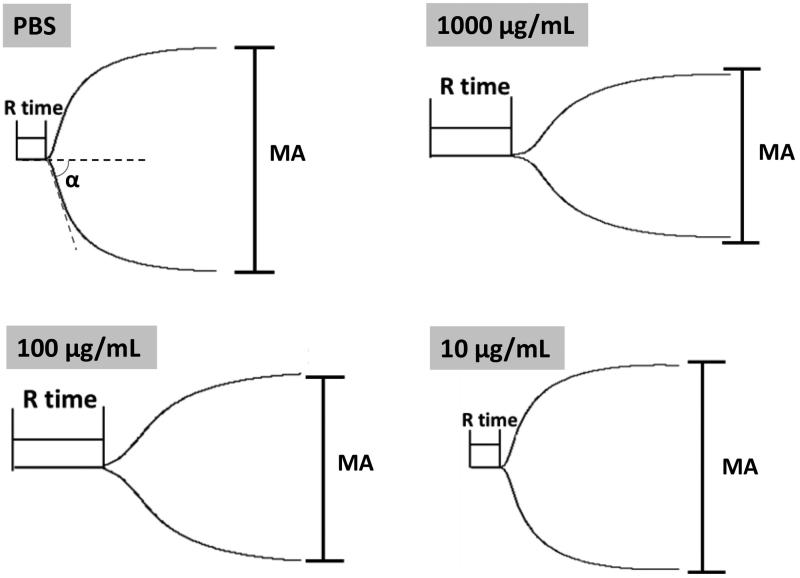
Representative TEG traces of whole blood coagulation in the presence of different concentrations of Tf-PAAs-MTX/pMMP-9.

As reported, APTT and PT alone cannot always accurately reflect the biomaterial-induced anticoagulant activity (Yildirim et al., 2013). Further studies were carried out by TEG test to provide the details of the blood clotting. TEG characterizes the formation and strength of the blood clot as a function of time and indicates some principle parameters in the blood clotting process: (1) reaction time *R*, time to reach 2-mm amplitude in the tracing, is the time from initiating the test to the initial fibrin formation; (2) coagulation time *K*, the period from 2 to 20 mm amplitude, represents the dynamics of clot formation; (3) *α* angle, the slope of the tangent joining the point of the 2-mm split intercepting the tracing, is the rate of clot aggregating or fibrin cross-linking; (4) maximum amplitude (MA) of the tracing, represents the maximum clot strength (Fan et al., 2018). Here, we measured the influence of Tf-PAAs-MTX/pMMP-9 on the whole blood clotting process, with the principle TEG data listed in Table 1. Additionally, the representative TEG traces were also given in Figure 8, showing the clot formation in PBS and Tf-PAAs-MTX/pMMP-9 at a concentration range of 10–1000 μg/mL. As shown, the initial straight line is tracing for a period time. At the end of the line, the trace turns into two curve lines. The amplitude between the two curve lines represents the clot strength. The period from the initial straight line to reach a 2-mm amplitude is the *R* time, and the period from 2 to 20 mm amplitude is the *K* time, representing the dynamics of clot formation. The slope of the point at the 2-mm amplitude is *α* angle. The two curve lines turns parallel and constant at last, showing the maximum amplitude (MA) of the tracing, this represents the maximum clot strength. From these results, characteristic TEG trace and normal clot parameters were obtained when clotting was carrying out in PBS or Tf-PAAs-MTX/pMMP-9. It was seen that Tf-PAAs-MTX/pMMP-9 at the concentration of 10 μg/mL showed no difference in TEG trace with the PBS control, and the main parameters were in normal range, suggesting excellent blood compatibility. When the concentration reached up to 100 or 1000 μg/mL, all parameters changed which implied that the clotting time prolonged and the clot strength decreased. These may be attributed from the cytotoxicity of MTX and the positive charges of the complexes. TEG assay is a sensitive analysis on the interactions between polymers and the proteins in the blood. As a cationic polymer, PAAs indeed interacted with the proteins in the blood, although the interaction was much weaker than other polymer such as PEI-25k. The weak interaction then induced the abnormal in some parameters in TEG assay at the high concentration of PAAs. However, due to the sensitized chemotherapy effect, the used concentration of Tf-PAAs-MTX in this work was not up to 100 μg/mL, so our drug delivery system showed relative good safety.

**Table 1.** Values of clotting kinetics for human whole blood mixed with Tf-PAAs-MTX/pMMP-9 solutions at different concentrations (*n* = 3).

Samples	*R* (min)	*K* (min)	*α* (deg)	MA (mm)
Normal range	5–10	1–3	53–72	50–70
PBS control	5.2	1.8	65.6	62.8
1000 μg/mL	14.5↑	5.6↑	34.9↓	49.1↓
100 μg/mL	16.7↑	5.0↑	37.1↓	49.0↓
10 μg/mL	5.9	1.9	63.0	61.2

As an injectable nanoparticle drug delivery system, its blood compatibility assays have been performed and confirmed that Tf-PAAs-MTX showed the good compatibility. Its good blood compatibility be attributed from its hyperbranched structure of PAAs segments and its low cationic charge density, which resulted in it showed the relative low zeta potential than other cationic polymer such as PEI-25k, and then was avoided to interact with the proteins in the blood (Tang et al., 2018).

**Figure 9.**
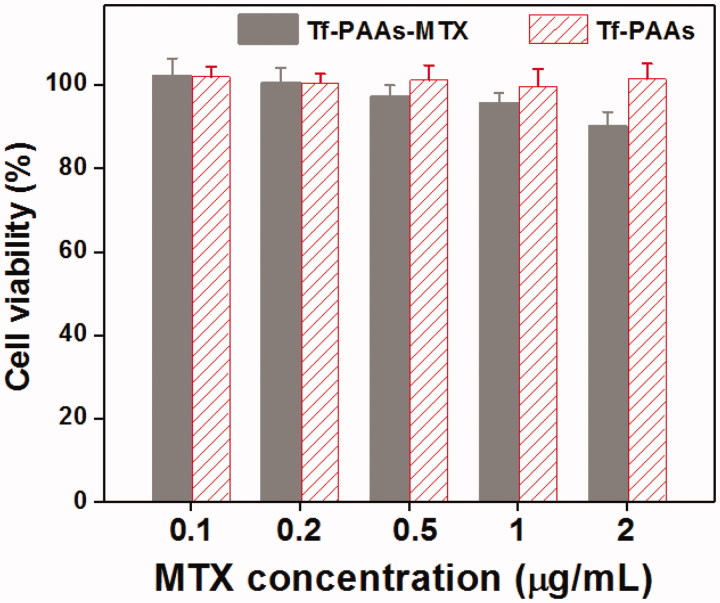
Cytotoxicity of Tf-PAAs and Tf-PAAs-MTX to 3T3 cells (37 °C and 5% CO_2_, *n* = 5).

The cytotoxicity of the blank Tf-PAAs and Tf-PAAs-MTX were also evaluated using 3T3 cells to confirm its safety, and the result was shown in Figure 9. It was found that the cell viability of the 3T3 cells treated with different concentration of Tf-PAAs or Tf-HPAA-GO for 24 h remained constant and there was no significant difference with PBS control. Even the concentration of Tf-HPAA-GO reached to 2 µg/mL, the 3T3 was still viable and no significant difference was found compared with PBS control. This result indicated that Tf-PAAs and its polymeric prodrug of Tf-HPAA-GO showed good safety.

**Figure 10.**
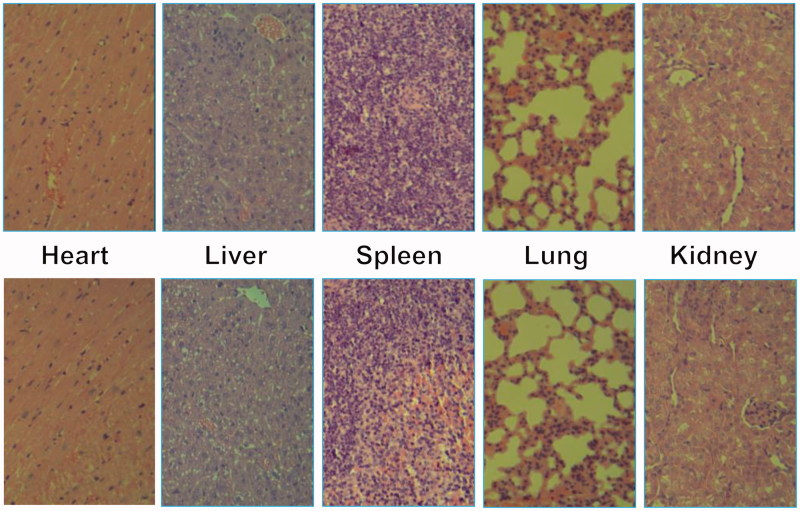
Representative HE stain image of organ histology by Tf-HPAA-MTX/pMMP-9 (top row) and PBS control (bottom row).

*In vivo* toxicity assay was also performed through a histological analysis to prove the safety of Tf-PAAs-MTX/pMMP-9. As shown in Figure 10, histologically, no visible difference was observed between the two groups. The *in vivo* toxicity of polymers is influenced by the chemical structures, size, biodistribution and metabolism as well as the surface and terminal groups (Li et al., 2016). The non-observed toxicity of Tf-PAAS-MTX/pMMP-9 could be attributed to its hyperbranched molecular structure and the degradation ability, which could reduce the cytotoxicity of polymers compared with a linear structure having the similar molecular weights.

## 4. Conclusion

A targeting polymeric prodrug to HNE-1 cells of Tf-PAAs-MTX was synthesized and then delivery pMMP-9 to form the drug and gene co-delivery system of Tf-PAAs-MTX/pMMP-9 in this work. This co-delivery system showed the enhanced chemotherapy to HNE-1 cells due to the consumption of intracellular GSH derived from the degradation of PAAs segments. Based on this, pMMP-9 improved further the inhibition effect to HNE-1 cells. This system could deliver pMMP-9 efficiently to HNE-1 cells in the presence of serum and induce the significant cell apoptosis as well as inhibit cell migration. The co-delivery of MTX and pMMP-9 showed the combined inhibition effect to HNE-1 cells *in vitro* and *in vivo*. Besides that, the co-delivery system showed good biocompatibility, including low cytotoxicity, slight interactions with blood component, low *in vivo* toxicity etc. This polymeric prodrug provided an easily delivery system combining the drug/gene co-delivery, chemotherapeutic sensibilization and targeting into one single platform, which showed a promising application in nasopharyngeal carcinoma therapy.
